# Haematological analysis of Japanese macaques (*Macaca fuscata*) in the area affected by the Fukushima Daiichi Nuclear Power Plant accident

**DOI:** 10.1038/s41598-018-35104-0

**Published:** 2018-11-13

**Authors:** Yusuke Urushihara, Toshihiko Suzuki, Yoshinaka Shimizu, Megu Ohtaki, Yoshikazu Kuwahara, Masatoshi Suzuki, Takeharu Uno, Shiori Fujita, Akira Saito, Hideaki Yamashiro, Yasushi Kino, Tsutomu Sekine, Hisashi Shinoda, Manabu Fukumoto

**Affiliations:** 1 Institute of Development, Aging and Cancer, Tohoku University, Miyagi, Japan; 2 Department of Radiation Biology, Tohoku University, Miyagi, Japan; 3 Graduate School of Dentistry, Tohoku University, Miyagi, Japan; 4 Research Institute for Radiation Biology and Medicine, Hiroshima University, Hiroshima, Japan; 5 Faculty of Medicine, Tohoku Medical and Pharmaceutical University, Miyagi, Japan; 6 Institute for Disaster Reconstruction and Regeneration Research, Tohoku University, Miyagi, Japan; 7 Tohoku Wildlife Management Center, Miyagi, Japan; 8 Department of Molecular Pathology, Tokyo Medical University, Tokyo, Japan; 9 Graduate School of Science and Technology, Niigata University, Niigata, Japan; 10 Department of chemistry, Tohoku University, Miyagi, Japan; 11 Institute for Excellence in Higher Education, Tohoku University, Miyagi, Japan

## Abstract

Several populations of wild Japanese macaques (*Macaca fuscata*) inhabit the area around Fukushima Daiichi Nuclear Power Plant (FNPP). To measure and control the size of these populations, macaques are captured annually. Between May 2013 and December 2014, we performed a haematological analysis of Japanese macaques captured within a 40-km radius of FNPP, the location of a nuclear disaster two years post-accident. The dose-rate of radiocaesium was estimated using the ERICA Tool. The median internal dose-rate was 7.6 μGy/day (ranging from 1.8 to 219 μGy/day) and the external dose-rate was 13.9 μGy/day (ranging from 6.7 to 35.1 μGy/day). We performed multiple regression analyses to estimate the dose-rate effects on haematological values in peripheral blood and bone marrow. The white blood cell and platelet counts showed an inverse correlation with the internal dose-rate in mature macaques. Furthermore, the myeloid cell, megakaryocyte, and haematopoietic cell counts were inversely correlated and the occupancy of adipose tissue was positively correlated with internal dose-rate in femoral bone marrow of mature macaques. These relationships suggest that persistent whole body exposure to low-dose-rate radiation affects haematopoiesis in Japanese macaques.

## Introduction

The Great Japan Earthquake and subsequent tsunami of March 2011 caused the Fukushima Daiichi Nuclear Power Plant (FNPP) accident, which released large amounts of artificial radioactive substances into the environment^1^. After people were evacuated, wild animals inhabiting the area became contaminated with artificial radionuclides^2^. These animals were exposed to long-term irradiation with a low-dose-rate of internally and externally deposited radionuclides. Seven years have passed since the FNPP disaster, but the biological effect caused by long-term exposure to radioactive caesium, a persistent nuclide, remains a major concern. Various biological impacts have been reported after the FNPP accident^3–6^. In some studies, the estimations of dose and dose-rate were carried out and were associated with the effect of wild life and livestock^7–12^. However, the extent to which radiation exposure is contributing to these findings remains unclear, due to the uncertainty attributed to fieldwork that is not controlled^13,14^.

Japanese macaques are a species of non-human primate with a life span of more than 20 years in a wild state. They form troops comprised of several dozen individuals with a mean home range size of 8.99 km^2^ (0.29–39.7 km^2^), irrespective of evergreen or deciduous habitat^15^. Japanese macaques are omnivorous and feed on plant leaves, fruits, insects and other small animals^16^. Those living in the Fukushima prefecture become sexually mature at around 5 years old^17^. Several troops of macaques had settled in certain areas around FNPP before the accident^18^, making them suitable subjects for determining the effect of long-term exposure to low-dose-rate radiation on humans. Leucocyte count in peripheral blood has been reported to have a significant inverse correlation with the muscle radiocaesium concentration in immature macaques captured in Fukushima city, located approximately 70 km northwest of FNPP^19^.

Haematopoiesis is one of the main vital processes in the body of mammals and is one of the most radiosensitive systems^20^. Bone marrow is the primary haematopoietic tissue in mammals, given that it produces all blood cells. Caused by moderate to high levels of radiation, acute whole-body irradiation reduces bone marrow cellularity and blood cell count^21,22^. The haematopoietic system is highly sensitive to radiation; whether at a large or medium dose, acute exposure damages its function^23^. However, the effect of long-term exposure to low-dose-rate radiation on the haematopoietic system, especially that of bone marrow, remains to be elucidated.

In this study, we collected haematological data from 95 Japanese macaques captured within a 40-km radius of FNPP (the exposed group) and within a range of 60-km to 100-km of FNPP (the non-exposed group). We estimated internal and external radiocaesium dose-rates using the ERICA Tool, and performed multiple regression analyses to evaluate the dose-rate dependency on haematological values, adjusting for possible background (confounding) covariates. While the environmental dose-rate from radionuclides in the soil is approximately 0.33 mSv/year in Japan^24^, the air dose-rate of several areas inhabited by the exposed group in this study was more than 100 times higher than the general background level^25^; therefore, haematological analysis of macaques inhabiting the area affected by the FNPP accident would provide insight into the effect of persistent exposure to low-dose-rate radiation on humans.

## Results

### Dose-rate and haematological values in peripheral blood

**Figure 1:**
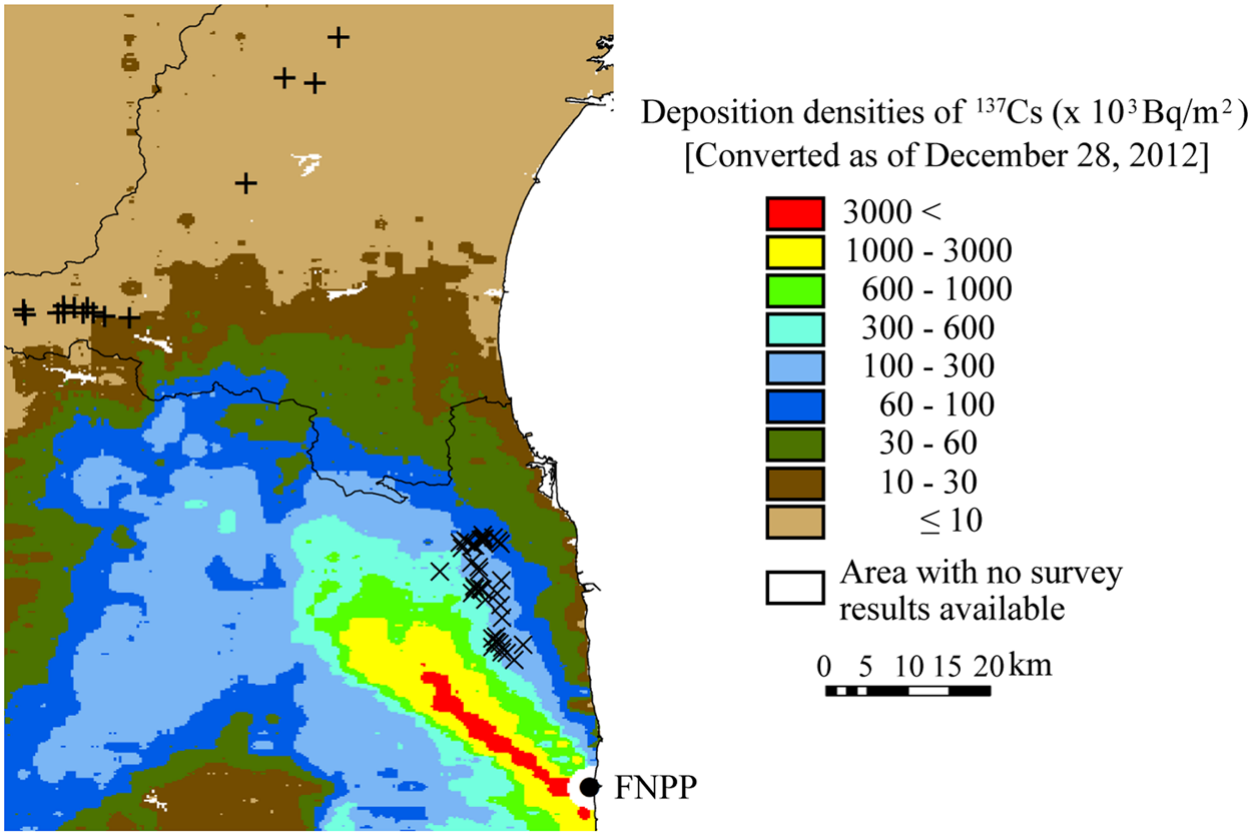
Map of the sampling point. The black circle indicates the location of Fukushima Daiichi Nuclear Power Plant (FNPP). X-marks and cross-marks indicate the sampling points of Japanese macaques in the exposed and the non-exposed areas, respectively.

We obtained peripheral blood samples from 42 exposed and 23 non-exposed Japanese macaques (Fig. 1). The response of chemokine and cytokine family genes to gamma irradiation is reportedly different between infant and adult mouse bone marrow tissues^26^. Ochiai, *et al*., previously divided Japanese monkeys into immature (0–4 years) and mature ($\geqq$5 years) groups^19^. Therefore, we adopted their classification in this study. The haematological values of peripheral blood are shown in Table 1. In members of the exposed group, the median radiocaesium (^134^Cs + ^137^Cs) activity concentrations in mature and immature macaques were 2,250 Bq/kg (ranging from 285 to 34,600 Bq/kg) and 1,280 Bq/kg (ranging from 376 to 24,500 Bq/kg), respectively, in femoral muscle. In members of the non-exposed group, the concentrations were 72.3 Bq/kg (ranging from 36.9 to 269.5 Bq/kg) for mature macaques and 57.3 Bq/kg for only one immature macaque. Red blood cell (RBC) count and hematocrit (Hct) were significantly lower in mature macaques of the exposed group than those of the non-exposed group.

**Table 1:** Haematological values in peripheral blood.

	Exposed group*						Non-exposed group**					
	Mature			Immature			Mature			Immature		
	N	Mean	SD	N	Mean	SD	N	Mean	SD	N	Mean	SD
WBC (×10^3^/mL)	19	9.3	5.9	23	12.2	6.4	22	9.8	3.1	1	12.8	N/A
RBC (×10^6^/mL)	19	4.5^††^	1.0	23	4.4	1.0	22	5.3	0.7	1	4.76	N/A
Hb (g/dL)	16	11.2	2.7	19	11.5	2.4	22	12.2	2.6	1	9.9	N/A
Hct (%)	16	38.7^†^	6.3	19	37.7	8.4	22	42.9	5.5	1	37.1	N/A
PLT (×10^3^/mL)	19	305.6	309.6	23	301.3	148.6	22	262.8	103.1	1	319	N/A
Radiocaesium concentration (^134^Cs + ^137^Cs, Bq/kg)	17	6200^†^	8780	22	3110	4990	14	102.7	71.8	1	76.3	N/A
Altitude^***^	16	70.9^†††^	22.2	23	77.4	33.6	22	384.7	94.2	1	404.4	N/A

^134^Cs and ^137^Cs activity concentration was corrected as of capture date.

WBC: White blood cells, RBC: Red blood cells, Hb: Hemoglobin, Hct: Hematocrit, PLT: Platelets.

^*^Exposed group includes troops inhabiting Fukushima prefecture (Minamisoma city and Iitate village).

^**^Non-exposed group includes troops inhabiting Miyagi prefecture (Sendai city and Kawasaki and Shichikasyuku towns).

^***^Data source is Digital elevation model (DEM) according to an open source from the Geospatial Authority of Japan^49^.

^†^Indicates significant difference from mature macaques of the non-exposed group (^†^0.01 ≤ p < 0.05, ^††^0.001 ≤ p < 0.01, ^†††^p < 0.001).

Using the ERICA Tool, we calculated internal and external dose-rates from the concentration of combined ^134^Cs and ^137^Cs (radiocaesium) in skeletal muscle and in soil, respectively (Table 2). The body size is different between mature and immature macaques, affecting the determination of dose conversion coefficient. Therefore, we estimated the dose-rate from dose conversion coefficients of four sizes of spheroids, as described in the Methods section. Minor and major axes of each spheroid were calculated by the plots of body length and body weight of 65 macaques (Supplementary Fig. S1a). According to the home range size of Japanese macaques^15^, we calculated an average ^137^Cs concentration in soil within a 3-km radius (corresponding to the area of a 30-km^2^ circle) from the capture point based on the mesh data of radiocaesium deposition in soil by the airborne monitoring survey^27^. ^137^Cs concentration in soil of the capture point showed significantly positive correlation with mean ^137^Cs concentration in soil within 30-km^2^ around the capture point (*N* = 79, r = 0.97, p < 0.001, Supplementary Fig. S2). We therefore propose that the external dose-rate calculated from that of the capture point in this study can be used as real external dose-rate. The radioactivity concentrations of radiocaecium in soil collected from the non-exposed area on December 28, 2012 were lower than 10,000 Bq/m^2^, which was undetectable by the airborne survey^27^. On the other hand, radiocaesium was detected in the skeletal muscle of members of the non-exposed group. The median internal dose-rate in the non-exposed group was 0.45 μGy/day (0.24–1.73 μGy/day). The median internal dose-rate was 7.6 μGy/day (1.8–219 μGy/day) and the external dose-rate was 13.9 μGy/day (6.7–35.1 μGy/day) in the exposed group. A weak positive correlation was found between external and internal dose-rates in the same individual (*N* = 69, r = 0.38, p < 0.001), whereas the combined internal and external (total) dose-rate was strongly associated with internal dose-rate (*N* = 69, r = 0.98 and p < 0.001, Supplementary Fig. S3).

**Table 2:** Estimated dose-rates of Japanese macaques (μGy/day).

Dose-rate	Exposed group		Non-exposed group	
	Median (Min – Max)	Mean ± SD	Median (Min – Max)	Mean ± SD
Internal	7.6 (1.8–219)	25.5 ± 38.3	0.45 (0.24–1.73)	0.64 ± 0.45
External	13.9 (6.7–35.1)	15.9 ± 8.0	<1.1^†^	<1.1^†^
Total	22.0 (8.8–231)	41.5 ± 42.1	N/A^†^	N/A^†^

^†^Estimated from the detection limit of the airborne survey (10,000 Bq/m^2^ of ^134^Cs and ^137^Cs)^27^.

We performed multiple regression analyses to evaluate dose-rate effects on haematological values in the peripheral blood of macaques, adjusting for the effect of confounding covariates such as sex, age, season of capture, and altitude of the capture point (adjusted by every 100 m). A significant contribution of internal dose-rate (a tendency to decrease with dose-rate) was detected in white blood cell (WBC) count and platelet (PLT) count in mature macaques. On the contrary, no correlation was observed between external dose-rate and any variable (Table 3 and Supplementary Table S1).

**Table 3:** Estimated coefficients of dose-rate effects on the haematological values in peripheral blood of macaques using multiple regression with covariates sex, age, season of capture date, and altitude of capture.

	*N* = *N*_1_*+N*_2_	Immature (*N*_1_)/Mature(*N*_2_)	Internal			External		
			coeff.	s.e.	p-value	coeff.	s.e.	p-value
WBC	53	Immature (23)	−0.00256	0.00183	0.168	0.00309	0.00471	0.516
		Mature (30)	−0.00211	0.00096	0.034*	−0.00246	0.00435	0.574
RBC	53	Immature (23)	0.00109	0.00073	0.139	−0.00292	0.00223	0.196
		Mature (30)	−0.00049	0.00039	0.214	−0.00254	0.00175	0.154
Hb	47	Immature (20)	0.00065	0.00072	0.371	−0.00093	0.00243	0.704
		Mature (27)	−0.00074	0.00038	0.060	0.00305	0.00254	0.238
Hct	47	Immature (20)	−0.00050	0.00065	0.452	0.00032	0.00220	0.884
		Mature (27)	−0.00023	0.00035	0.526	0.00336	0.00240	0.168
PLT	53	Immature (23)	−0.00314	0.00268	0.247	−0.00079	0.00699	0.911
		Mature (30)	−0.00452	0.00144	0.003**	0.00086	0.10700	0.994

Each set of regression data with sample size (N) consists of two subsets: “Immature” and “Mature”. Multiple regression was performed without separating these subsets.

WBC: White blood cells, RBC: Red blood cells, Hb: Hemoglobin, Hct: Hematocrit, PLT: Platelets, coeff.: coefficient, s.e.: standard error.

*0.01 ≤ p < 0.05, **0.001 ≤ p < 0.01.

### Dose-rate and haematopoietic cells in bone marrow

**Figure 2:**
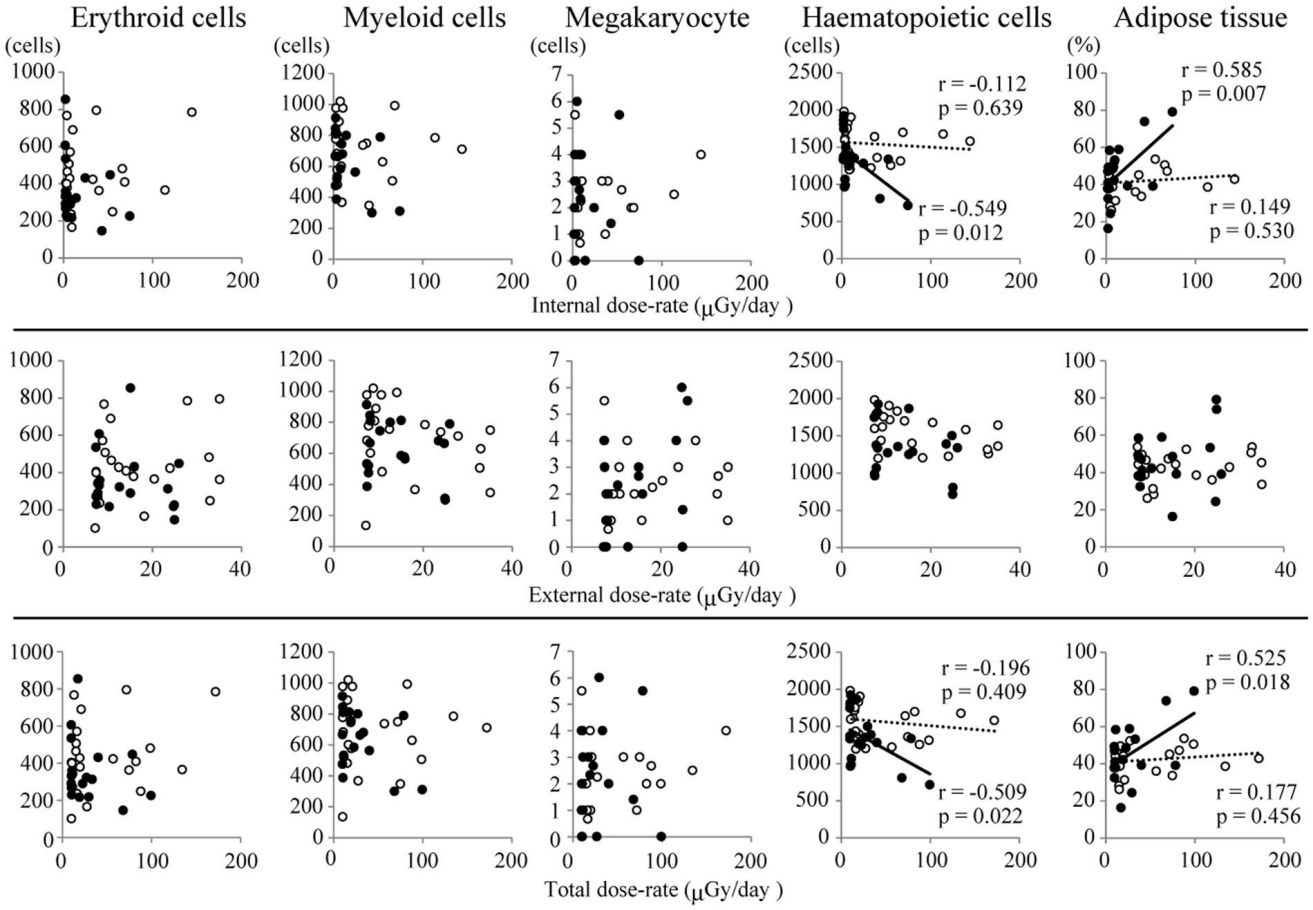
Correlation between dose-rate and haematological values in bone marrow of the exposed group. White circles: immature macaques (N = 20), black circle: mature macaques (N = 18). Cell number is presented per 115,600 μm^2^ of bone marrow. Solid lines and dashed lines indicate linear trend lines which were drawn by the scatter plots of mature and immature animals, respectively. r and p indicate Pearson’s correlation coefficient and p value, respectively. All Pearson’s correlation coefficients and p values are shown in Supplementary Table S2.

**Figure 3:**
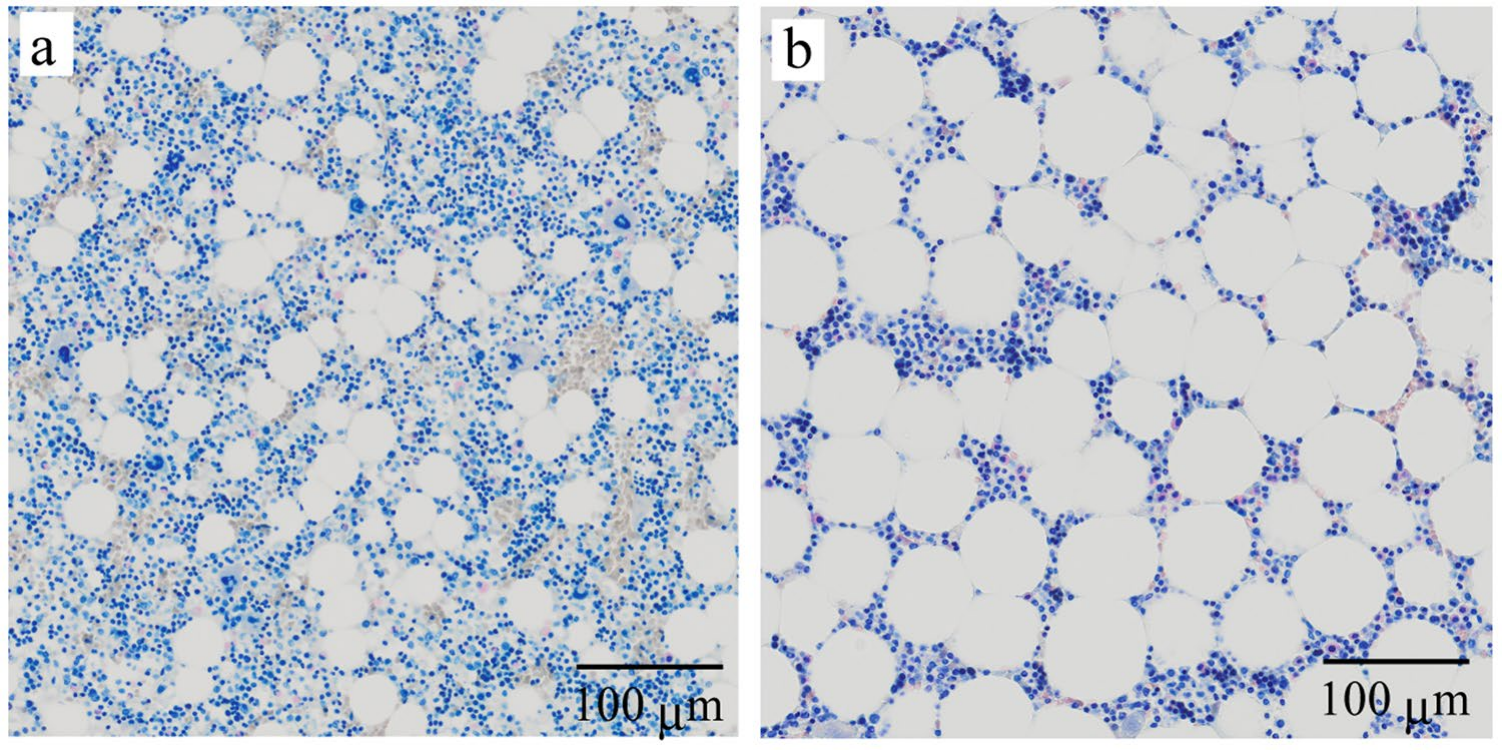
Representative histology of bone marrow of the exposed macaques. (**a**) A 9-year-old male captured on August 27, 2013 with 479 Bq/kg (^134^Cs + ^137^Cs) of the skeletal muscle, of which the estimated internal dose-rate was 4.90 μGy/day and the external dose-rate was 24.8 μGy/day. (**b**) An 8-year-old female captured on January 24, 2014 with 11,400 Bq/kg (^137^Cs + ^134^Cs) of the skeletal muscle, of which the estimated internal dose-rate was 74.5 μGy/day and the external dose-rate was 24.9 μGy/day.

We obtained bone marrow samples from the femur of 18 mature and 20 immature macaques of the exposed group. We counted the cell number of each haematopoietic cell lineage in sampled bone marrow and analyzed a correlation of haematopoietic cells and dose-rate (Figs 2 and 3; Supplementary Table S2). Using similar methods, we also performed multiple regression analyses to examine dose-rate effects on haematopoietic values in bone marrow of macaques (Table 4 and Supplementary Table S3). In mature macaques, significant inverse correlations with internal dose-rate were detected in myeloid, megakaryocyte, and haematopoietic cell counts. In addition, the opposite tendency was detected in adipose tissue from bone marrow of mature macaques. On the other hand, no consistent correlation was observed between external dose-rate and haemotological values.

**Table 4:** Estimated coefficients of dose-rate effects on the haematological values in bone marrow using multiple regression with covariates sex, age, season of capture date, and altitude of capture.

	*N* = *N*_1_ + *N*_2_	Immature (*N*_*1*_)/Mature (*N*_*2*_)	Internal			External		
			coeff.	s.e.	p-value	coeff.	s.e.	p-value
Erythroid cells	38	Immature(20)	0.00117	0.0013	0.373	−0.003	0.00491	0.542
		Mature (18)	−0.00268	0.00309	0.393	0.0018	0.00858	0.853
Myeloid cells	38	Immature(20)	0.00142	0.00083	0.099	−0.0098	0.00315	0.004**
		Mature (18)	−0.00506	0.00198	0.016*	0.00656	0.00550	0.242
Megakaryocytes	38	Immature(20)	0.00024	0.00139	0.865	0.00017	0.00512	0.974
		Mature (18)	−0.00717	0.00335	0.040*	0.0212	0.00944	0.032*
Haematopoietic cells	38	Immature(20)	0.00049	0.00056	0.386	−0.0042	0.00211	0.055
		Mature (18)	−0.00493	0.00133	0.001***	0.0057	0.00370	0.131
Adipose tissue	38	Immature(20)	0.00005	0.00078	0.950	−0.0021	0.00357	0.570
		Mature (18)	0.00672	0.00185	0.001**	−0.0164	0.00571	0.007**

Each set of regression data with sample size (*N)* consists of two subsets: “Immature” and “Mature”. Multiple regression was performed without separating these subsets.

coeff.: coefficient, s.e.: standard error.

*0.01 ≤ p < 0.05, **0.001 ≤ p < 0.01, ***p < 0.001.

## Discussion

Total dose-rate was highly associated with internal dose-rate (Supplementary Fig. S3), suggesting that the quantified effects found in this study reflect internal dose-rate. The soil radiocaesium concentration from the capture points showed a significantly positive correlation with mean radiocaesium concentration in soil within 30 km^2^ around the capture point (Supplementary Fig. S2). The ratio of the mean radiocaesium concentration in soil within 30 km^2^ from the capture point to the radiocaesium concentration of capture point was 1.12 ± 0.18 (S.D.), ranging from 0.92 to 1.57. We consider the error of the calculated external dose-rate to be within an allowable range. However, external dose-rate was not measured directly for each individual. In comparison with the internal dose-rate, more uncertainty may be mediated in the calculation of external dose-rate in this study.

It is important to investigate the living conditions and temporal dose-rate changes of macaques, in order to determine whether or not low-dose-rate radiation affects haematopoiesis. The variance of internal dose-rate was larger than that of external dose-rate in the exposed group (Table 2). Japanese macaques are omnivorous^16^. The variance of internal dose-rate in insects and small animals has been reported to be greater than the external dose-rate variance^28^. These data suggest that the variance of radiocaesium concentration in Japanese macaques is reflected by that of their prey. In our previous study, we analyzed the correlation of oxidative stress markers in calf peripheral blood with either internal or external dose, or with internal or external dose-rate. We found that oxidative stress status is closely related to internal dose-rate but to no other conditions^11^. We estimated the whole body dose-rate using the ERICA Tool and the haematopoietic cell count in bone marrow and correlated with the internal dose-rate (Table 4 and Supplementary Table S3). Various studies have reported that radiocaesium concentration in organs, including peripheral blood, is highly correlated with that in skeletal muscles in animals around FNPP after the accident^29,30^. Haematopoietic bone marrow is surrounded by skeletal muscles. Radiocaesium concentration tends to be highest in skeletal muscles, more than 20-fold that of peripheral blood. Furthermore, skeletal muscles compromise most of the body weight. Taken together, these suggest that internal dose-rate of bone marrow is derived from skeletal muscles, which can be estimated using radiocaesium concentration of femoral muscle. However, it is important to measure radionuclides in each organ for estimation of marrow dose-rate.

We chose populations of macaques residing at the same latitude and in adjacent prefectures to ensure that the environments were as identical as possible. Wild Japanese macaques may be infected with various pathogens. This can result in modest to marked effects on the intestine and its physiologic and immunologic functions, including peripheral blood cell count, so we carefully chose the members of the non-exposed group. We did not observe any macroscopic differences between the exposed and the non-exposed macaques, including coat and the amount of subcutaneous fat which are both affected by climate, nor did we observe any anomaly during dissection. Compared with the non-exposed group, the exposed group showed a lower RBC count and Hct, but these were still within the normal ranges. The mean altitude of capture points in the exposed group was significantly lower than that of the non-exposed group (Table 1). The natural environment of the exposed group may be different from that of the non-exposed group. Therefore, to exclude the effect of confounding covariates like sex, age, and the condition of capture, we applied multiple regression analyses and adjusted for possible effects of the confounders to evaluate dose-rate effects on the haematological values in peripheral blood. WBC and PLT counts in the peripheral blood of mature macaques were revealed to be inversely correlated with internal dose-rate (Table 3 and Supplementary Table 1). It should be noted, however, that the present study was not performed under strictly controlled conditions, but used wild macaques, and we could not therefore rule out environmental factors as confounders. Data collected from macaques, a closely-related species to humans, will contribute to radiation protection in humans. Therefore, it is necessary to vigilantly monitor both exposed and non-exposed macaque groups over time to elucidate the ultimate effect of very low-dose-rate radiation on the haemopoietic system, which may indicate a similar response in humans.

The haematopoietic system is one of the most radiosensitive tissues in the body^20^. Bone marrow is composed of haematopoietic cells and adipocytes and radiation suppresses the haematopoietic system, leading to increased concentrations of adipocytes. Total lipids of rat bone marrow reach maximum levels 1 week after exposure to radiation as a function of dose, but total lipids slowly decrease with time. Similarly, 8 days after 8-Gy irradiation, monkeys show a decrease in total marrow^31^. In this study, the ratio of the occupancy area of haematopoietic cells to adipocytes in bone marrow of mature macaques was positively correlated with internal dose-rate (Table 4 and supplementary Table S3). This shows that persistent exposure to low-dose-rate radiation, as well as acute high-dose radiation, is toxic to haematopoietic systems. The haematopoietic system is organized in a hierarchical manner. For example, damaged multipotent progenitors and haematopoietic progenitor cells result in myelosuppression, which is consistent with the suppression of all lineages of haematopoietic cells. In mature macaques, myeloid cell, megakaryocyte, and haematopoietic cell counts in bone marrow were inversely correlated with internal dose-rate (Table 4 and Supplementary Table S3). Furthermore, WBC and PLT counts in peripheral blood were inversely correlated with internal dose-rate (Table 3 and Supplementary Table S1). However, WBC and PLT counts in peripheral blood showed no significant difference between exposed group and non-exposed group (Table 1). Adult C57BL/6 mice exposed to gamma-ray radiation show a significantly decreased formation of mixed types of colonies of haematopoietic/progenitor cells in bone marrow, even under daily exposure to 10 mGy for 1 month^32^. Being exposed to 22.6 μGy/h of gamma-radiation for 2.5 years, free-ranging meadow vole populations have higher counts of neutrophils in peripheral blood than either the controls or high-dose voles (3,840 μGy/h) but have lower hematocrit than the controls^33^. We recently reported that spermatogenesis was accelerated in large Japanese wild mice residing the ex-evacuation zone of the FNPP accident, but sperm number remained constant^12^. These findings suggest that the compensatory mechanism in haematopoiesis maintains homeostasis under persistent exposure to very low-dose-rate radiation and that radiation effects are different between laboratory animals and field animals. We need to continue to carefully observe and interpret the biological effects induced by long-term exposure to very low-dose-rate radiation.

Ochiai, *et al*., reported that WBC counts in peripheral blood are inversely correlated with radiocaesium activity concentrations in the muscle, even though the mean values are lower than 1,000 Bq/kg in immature macaques captured in Fukushima city^19^. In our study, radioactivity concentrations of ^137^Cs in the femoral muscle of immature macaques of the exposed group ranged from 308 to 24,500 Bq/kg (median 1,200 Bq/kg), which is a remarkably high level of internal radiocaesium compared with their study. In addition, we estimated internal and external dose-rates and performed multiple regression analyses for the dose-rate dependence of haematological values. However, we did not observe an association between any haematological value in peripheral blood and internal dose-rate in immature macaques (Table 3 and Supplementary Table S1). We previously reported that, based on the γH2AX foci, cattle from the ex-evacuation zone had significantly higher levels of DNA damage in lymphocytes compared to levels in non-affected cattle^34^. However, the damage levels gradually decreased from 500 to 700 days after the FNPP accident. Our sampling was performed between May 2013 and December 2014, but Ochiai, *et al*., collected samples between April 2012 and March 2013. The contradiction in findings may be caused by the difference in the sampling period.

Our study revealed that the myeloid cell, megakaryocyte, and haematopoietic cell counts in bone marrow were inversely correlated with the internal dose-rate in mature macaques of the exposed group. The main cause of myelosuppression by persistent exposure to very low-dose-rate radiation remains unclear. We previously reported that the level of oxidative stress was significantly correlated with the internal dose-rate of radiocaesium. The levels were less than 50 μGy/day in cattle captured from the ex-evacuation zone of the FNPP accident^11^. Hypersensitivity to irradiation has been reported in the haematopoietic stem cells (HSCs) of adult mice. Even irradiation of 0.02 Gy X-rays causes an immediate increase in reactive oxygen species (ROS). However, total body irradiation with 0.02 Gy does not decrease HSC numbers unless the HSC microenvironment is altered by an inflammatory insult^35^. Health effects of people exposed to persistent low-dose gamma-ray derived from Iridium-192 were studied, in which the subjects received 0.05–0.65 Gy for 72 days and, over the course of 10 years, various degrees of immune dysfunction and abnormalities of blood cells and bone marrow are identified. Participants recovered within 1–3 years of exposure^36^. Levels of Chernobyl liquidators at or above 200 mGy result in an increased risk of leukemia, after excluding smoking as a major confounding factor^37^. An international cohort study of nuclear workers reveal positive associations between chronic low-dose radiation exposure and excess relative risk of leukaemia mortality in nuclear workers exposed to very low-dose (mean 1.1 mGy/year) radiation. The mean follow-up period of the study is 27 years^38^. Ramsar, Iran is among the places in the world with the highest levels of natural radiation. This has had a non-detrimental effect on the people living there, who seem to have adapted to mean annual exposure levels of 10 (and up to 260) mGy/year^39,40^. This suggests that the effect of long-term very low-dose-rate radiation is a consequence of complicated biological responses. We therefore propose that continuous observation on the macaques inhabiting around FNPP is necessary to determine whether long-term exposure to low-dose-rate radiation induces irreversible myelosuppression, maintains basal haematopoiesis, or causes haematological malignancies. Cytogenetic dosimetry is recognized as a valuable assessment method in measuring acute exposure to more than 100 mGy of low linear energy transfer (LET) radiation^41^. Collaborative efforts are now underway to identify chromosome aberrations in peripheral blood lymphocytes of Japanese macaques for biological dose evaluation.

This study is the first to report that haematopoiesis may be adversely affected by low-dose-rate radiation in primates. To understand the human response to long-term exposure to very low-dose-rate radiation, Japanese macaques inhabiting the area affected by the FNPP accident are the most suitable wild animal, since they do not recognize or fear radiation and do not smoke, which is the most confounding factor in analyzing the effects of low-dose radiation on humans. Therefore, the present study provides extremely crucial data for understanding the effect of chronic, very low-dose-rate radiation on humans. Radiocaesium concentrations in the organs of macaques belonging to the exposed group have been continuously high, attributable to the intake of radiocontaminated foods. The life span of Japanese macaques is approximately 20 years, and many individuals born after the FNPP accident have been observed. Therefore, macaques inhabiting the exposed area are suitable to study in order to identify and measure the effects, including transgenerational effects, of chronic very low-dose-rate irradiation on humans. We should vigilantly monitor these populations of macaques over time.

## Methods

### Animals and Ethics statements

Japanese macaques were culled to prevent damage to crops according to the Japanese Monkey Management Plan based on the Wildlife Protection and Hunting Management Law. Macaques were captured using box traps and killed by licensed hunters at the request of each local government. The method of capture and killing was carried out according to the guidelines published by the Primate Research Institute of Kyoto University^42^. Japanese macaques inhabiting the areas of this study were not listed as endangered species on the Japanese Red List revised by the Ministry of Environment, Japan^43^.

This entire study was approved by the Institutional Animal Care and Use Committee of the Center for Laboratory Animal Research, Tohoku University (Approved number: 2014 IDAC-037). All experiments were performed in accordance with relevant guidelines and regulations. The macaques analyzed in this study were captured from May 2013 to December 2014. The exposed group consisted of 72 macaques (71 from Minamisoma city and 1 from Iitate village) in Fukushima prefecture. The non-exposed (control) group consisted of 23 macaques (17 from Shichikasyuku Town, 4 from Sendai City, and 2 from Kawasaki Town) in Miyagi prefecture (Fig. 1). We obtained 42 peripheral blood samples, 69 femoral muscles, and 38 femurs from the exposed group. From the non-exposed group, we collected 23 peripheral blood samples and 15 femoral muscles. The age of each macaque was determined by counting growth layers in the dental cementum^44^. The macaques were divided into 2 groups: the immature group (0–4 years) and the mature group ($\geqq$5 years) according to Ochiai, *et al*.^19^.

### Measurement of radioactivity concentration

Radioactivity was determined by gamma-ray spectrometry, specifically by using high-purity Germanium detectors, as described previously^29^. In brief, duration of the measurement varied from 3,600 to 86,400 seconds, depending on the radioactivity concentration of the sample. Standard volume sources of different sizes were prepared by diluting stock solutions of ^137^Cs and ^152^Eu and by gelling with a highly absorbent polymer. Samples were homogenized and scaled in a polyethylene tube. The nuclide was identified by its characteristic photopeaks (greater than 3σ above the baseline). Samples were stored at −30 °C until radioactivity measurements were taken. Therefore, the radioactivity concentration of radiocaesium was corrected to the capture date.

### Haematological analysis

Peripheral blood was collected from the hearts of the carcasses and was immediately mixed in a tube containing ethylenediaminetetraacetic acid disodium salt (EDTA-2Na). The numbers of WBC, RBC, and PLT, haemoglobin concentration (Hb), and Hct were measured with a full automatic blood cell counter (PCE-310, ERMA Inc., Tokyo, Japan).

We examined bone marrow from the femurs of 18 mature (8–10 years) and 20 immature macaques (2–4 years) of the exposed group. The femur was cut at its central part immediately after sampling, fixed in 4% formaldehyde, and decalcified in 10% EDTA-2Na at room temperature. Paraffin-embedded bone marrow tissue from the femur was sectioned and stained with Giemsa solution. The image of stained specimens was captured by a Virtual Slide System VS120-L100 (OLYMPUS, Tokyo, Japan). The cell number of each haematopoietic cell lineage, such as erythroid cells, myeloid cells, and megakaryocytes, was counted in bone marrow image of a 2000 pixel (170 nm/pixel) square. The occupancy of haematopoietic cells and adipose tissue was calculated using an open source image processing program, ImageJ^45^.

### Dose-rate estimation

Dose-rate by radiocaesium exposure in Japanese macaques was calculated using the ERICA Tool (version 1.2)^46^ with some modifications. The ERICA Tool’s default radiation weighting factors of 1 for β/γ-rays and 3 for low energy β-rays were used. The following conditions were used in this study: Tier 2 assessment, “Terrestrial”, “Mammal”, “Ground-living animal”, “On-soil,” and an occupancy factor value of 1.0. For the calculation of external dose-rate, the infinite plane isotopic source at 0.5 g/cm^2^ depth was defined as the radionuclide distribution patterns in soil. To determine dose conversion coefficients (DCCs) using the equation provided by the ERICA Tool, we used a spheroid to approximate the shape of the macaque’s body, with the body length spanning across the long axis. We plotted the body length and the body weight of 65 macaques. Assuming that the specific gravity of the body is 1.0, we calculated the body width, which was imputed as the minor axis of the equation. For the body length, we used 30 cm for individuals between 25 and 35 cm. Likewise, 40, 50, and 60 cm were used instead of the actual body length (Supplementary Fig. S1). DCCs were calculated and are shown in Supplementary Table S4.

Several studies have reported that radiocaesium activity concentration is the highest in the skeletal muscle among the organs measured in animals inhabiting areas near the Fukushima Daiichi Nuclear Power Plant (FNPP) accident^29,30^. Assuming that the whole body of the macaque was composed of skeletal muscle, the internal dose-rate was calculated using the radioactivity concentration of radiocaesium in the femoral muscle. Calculations of external dose-rate were based on deposited radiocaesium in soil, where Japanese macaques were captured. The available mesh data of radiocaesium deposited in soil, provided by the airborne monitoring survey, was closest temporally (December 28, 2012) to our study (May 2013 to December 2014)^27^. Using these mesh data, the radiocasesium deposit density was calculated and the external dose-rate was corrected, based on physical delay, to the sampling day. Total dose-rate indicates the sum of internal and external dose-rate.

### Statistical Analyses

Welch’s *t*-test was used to compare haematological values in peripheral blood between the exposed and non-exposed groups.

We performed multiple regression analyses for the dose-rate dependence of peripheral blood and bone marrow cells. As explanatory variables, we chose internal dose-rate, external dose-rate, age, sex, season of capture and altitude of the capture site (adjusted by every 100 m). The regression models can be elaborated as follows:

Given a set of data {(*Y*_*i*_, age_*i*_, mature, sex_*i*_, seaseon_*i*_, altitude_*i*_), *i* = 1, …, *n* (serial number of individual)}, we assumed the regression model was specified as:

$$\begin{array}{lll}{Y}_{i} & = & \mu +\{{\beta }_{1}\cdot (1\,-\,{{\rm{m}}{\rm{a}}{\rm{t}}{\rm{u}}{\rm{r}}{\rm{e}}}_{i})+{\beta }_{2}\cdot {{\rm{m}}{\rm{a}}{\rm{t}}{\rm{u}}{\rm{r}}{\rm{e}}}_{i}\}\cdot {x}_{Ii}+\{{\beta }_{3}\cdot (1\,-\,{{\rm{m}}{\rm{a}}{\rm{t}}{\rm{u}}{\rm{r}}{\rm{e}}}_{i})+{\beta }_{4}\cdot {{\rm{m}}{\rm{a}}{\rm{t}}{\rm{u}}{\rm{r}}{\rm{e}}}_{i}\}\cdot {x}_{Ei}\\  &  & +{\gamma }_{1}\cdot {{\rm{a}}{\rm{g}}{\rm{e}}}_{i}+{\gamma }_{2}\cdot {{\rm{s}}{\rm{e}}{\rm{x}}}_{i}+{\gamma }_{3}\cdot {{\rm{s}}{\rm{e}}{\rm{a}}{\rm{s}}{\rm{o}}{\rm{n}}}_{i}+{\gamma }_{4}\cdot {{\rm{a}}{\rm{l}}{\rm{t}}{\rm{i}}{\rm{t}}{\rm{u}}{\rm{d}}{\rm{e}}}_{i}+{\varepsilon }_{i},\,i=1,\,\ldots ,\,n,\end{array}$$

where *Y*_*i*_ denotes the logarithmic transformed observed value of the *i* th sample, age_*i*_ indicates age of “*i*” at capture, mature_*i*_ defined as mature_*i*_ = 1 if age_*i*_ ≥ 5.0, or 0, if age_*i*_ < 5.0, sex_*i*_ denotes sex of “*i*” (1:male, 0:female), and season_*i*_ is defined by season_*i*_ = 1, if the “*i*” was captured in the period from April to September, or 0, if the “*i*” was captured in the period from October to March, altitude_*i*_ denotes the sea level altitude of “*i*” at capture in 100 m units, *ε*_*i*_’s denote error terms, consisting of measurement error and individual variation, which are realizations of independent random variables from a normal distribution with mean 0 and variance *σ*^2^. The parameters μ, *β*_1_, …, *β*_4_, *γ*_1_, …, *γ*_4_ are unknown regression coefficients to be estimated, which represent the intercept (*μ*), dose-rate effect factor by internal exposure in immature individuals (*β*_1_), dose-rate effect factor by internal exposure in mature individuals (*β*_2_), dose-rate effect factor by external exposure in immature individuals (*β*_3_), dose-rate effect factor by external exposure in mature individuals (*β*_4_), and effects of the confounding background covariates concerning age (*γ*_1_), sex (*γ*_2_), season (*γ*_3_), and altitude (*γ*_4_), respectively.

The least squares method was applied for estimating the unknown parameters. Since the number of samples (*n*) is small, based on AIC (Akaike’s Information Criterion)^47^, we optimized the model with selecting variables of confounding background factor candidates. The R software (ver. 3.4)^48^ was used for implementing statistical data analyses. Using the parallel method with the same explanatory variables to the analyses of peripheral blood cells, we also performed multiple regression analyses for dose-rate dependency of bone marrow cells. We set our significance level at <0.05 for all statistical procedures.

## Electronic supplementary material

Supplementary Figures and Tables
