# Supplementary material for: Haematological analysis of Japanese macaques (Macaca fuscata) in the area affected by the Fukushima Daiichi Nuclear Power Plant accident
Source: Sci Rep. 2018 Nov 13;8:16748. doi: 10.1038/s41598-018-35104-0 (PMC6233195; doi:10.1038/s41598-018-35104-0)
Supplement: Supplementary file 1 — Supplementary Figures and Tables [file 41598_2018_35104_MOESM1_ESM.pdf]

Title

Haematological analysis of Japanese macaques (*Macaca fuscata*) in the area affected by the Fukushima Daiichi Nuclear Power Plant accident

Yusuke Urushihara[1,2], Toshihiko Suzuki[3], Yoshinaka Shimizu[3], Megu Ohtaki[4], Yoshikazu Kuwahara[5], Masatoshi Suzuki[6], Takeharu Uno[7], Shiori Fujita[3], Akira Saito[8], Hideaki Yamashiro[9], Yasushi Kino[10], Tsutomu Sekine[11], Hisashi Shinoda[3], Manabu Fukumoto[1, 8*]

[1]Institute of Development, Aging and Cancer, Tohoku University, Miyagi, Japan
[2]Department of Radiation Biology, Tohoku University, Miyagi, Japan
[3]Graduate School of Dentistry, Tohoku University, Miyagi, Japan
[4]Research Institute for Radiation Biology and Medicine, Hiroshima University, Hiroshima, Japan
[5]Faculty of Medicine, Tohoku Medical and Pharmaceutical University, Miyagi, Japan
[6]Institute for Disaster Reconstruction and Regeneration Research, Tohoku University, Miyagi, Japan
[7]Tohoku Wildlife Management Center, Miyagi, Japan
[8]Department of Molecular Pathology, Tokyo Medical University, Tokyo, Japan
[9]Graduate School of Science and Technology, Niigata University, Niigata, Japan
[10]Department of Chemistry, Tohoku University, Miyagi, Japan
[11]Institute for Excellence in Higher Education, Tohoku University, Miyagi, Japan

*Corresponding author
Manabu Fukumoto M.D., Ph.D.
Department of Molecular Pathology, Tokyo Medical University
6-1-1, Shinjuku, Shinjuku-ku, 1608402, Japan
Tel.: +8133516141
Fax: +8133526335
E-mail: manabu.fukumoto.a8@tohoku.ac.jp

Supplementary Figure S1

a

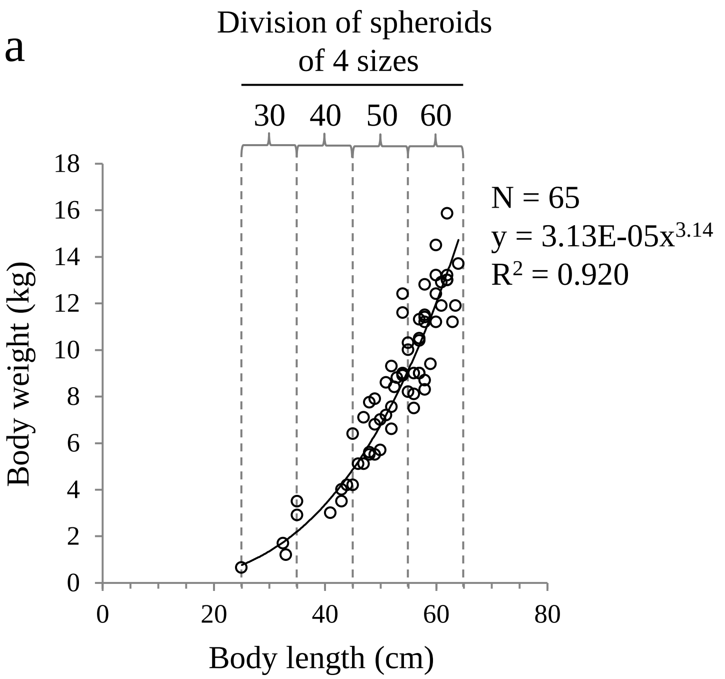

b

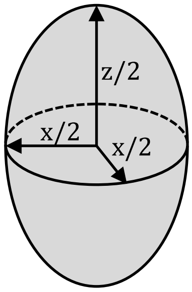

Assuming that the specific gravity of macaques is 1.0,
$v = 3.13E\text{-}02z^{3.14}$

$$v = \frac{4}{3}\pi\left(\frac{x}{2}\right)^2\frac{z}{2} = \frac{\pi x^2 z}{6}$$

$$x = \left(\frac{6v}{\pi z}\right)^{-2}$$

v: volume of the spheroid (cm^3)
x: minor axis (cm) = body width
z: major axis (cm) = body length

Supplementary Figure S1. Estimation of dose conversion coefficients.
a. The plot shows the correlation between body length and body weight of 65 exposed macaques. The curved line indicates an approximation curve. The dashed lines indicate borders of the spheroids of 4 sizes. R^2: coefficient of determination.
b. Assuming that the specific gravity of macaques is 1.0, the volume of spheroids was calculated from the major axis (Height) and minor axis (Width and Length).
To calculate dose conversion coefficients (DCCs), x and z were inserted into the ERICA Tool equation.

Supplementary Figure S2

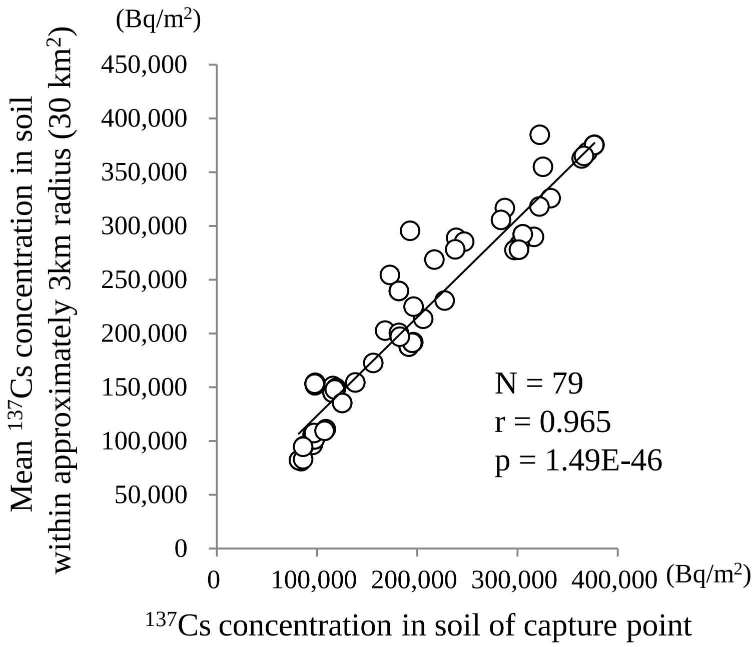

Supplementary Figure S2. Correlation between ^{137}Cs concentration in soil of capture point and mean ^{137}Cs concentration in soil within 30 km^2 around capture point.

The mean ^{137}Cs concentration in soil within approximately 3km radius (30 km^2) from capture point was calculated based on the mesh data of radiocaesium deposition in soil by the airborne monitoring survey[25]. Solid line indicates a linear trend line. ^{137}Cs concentration in soil of capture point is closely associated with mean ^{137}Cs concentration in soil within 30 km^2 around capture point. r and p indicates pearson's correlation coefficient and p values, respectively.

Supplementary Figure S3

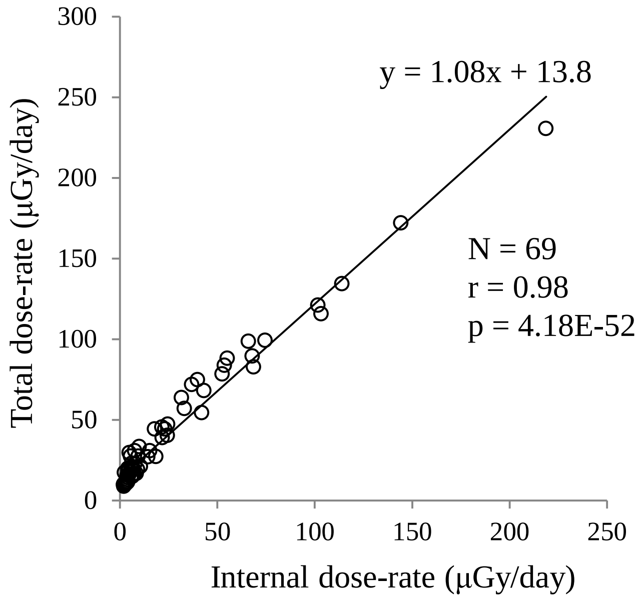

Supplementary Figure S3. Correlation between internal dose-rate and total dose-rate.
Total dose-rate, that is the combined dose-rate of the internal and external exposure is closely associated with the internal dose-rate. r and p indicates pearson's correlation coefficient and p values, respectively.

Supplementary Table S1

Supplementary Table S1. Results of multiple regression analyses for haematological values in the peripheral blood of macaques with covariates sex, age, season of capture date and altitude of capture : Estimated regression coefficients and goodness of fit using the full model and the optimized model.

WBC (full model)

	coeff.	s.e.	p-value	
internal x immature	-0.00225	0.00186	0.232	
internal x mature	-0.00204	0.000994	0.0463	*
external x immature	-0.00106	0.00596	0.8594	
external x mature	-0.00225	0.00558	0.545	
age	-0.00756	0.0107	0.483	
sex	-0.089	0.0636	0.169	
season	0.0975	0.0673	0.155	
altitude	-0.0128	0.0299	0.67	

N=53,Adjusted R^2=0.143,AIC=-1.638
F-statistic: 2.085 on (8, 44), p-value: 0.079

WBC (optimized model)

	coeff.	s.e.	p-value	
internal x immature	-0.00256	0.00183	0.168	
internal x mature	-0.00211	0.000964	0.0336	*
external x immature	0.00309	0.00471	0.516	
external x mature	-0.00246	0.00435	0.574	
age	-	-	-	
sex	-0.0833	0.0628	0.191	
season	0.0975	0.0667	0.151	
altitude	-	-	-	

N=53, Adjusted R^2=0.155, AIC=-4.0125
F-statistic: 2.587 on (6, 46), p-value: 0.0304*

RBC (full model)

	coeff.	s.e.	p-value	
internal x immature	0.00095	0.00075	0.208	
internal x mature	-0.00046	0.00040	0.253	
external x immature	-0.00217	0.0024	0.371	
external x mature	-0.00172	0.00225	0.448	
age	0.00597	0.0043	0.172	
sex	0.01990	0.0256	0.440	
season	0.05950	0.0271	0.0335	*
altitude	0.00787	0.0120	0.516	

N=53, Adjusted R^2=0.148, AIC=-98.080
F-statistic: 2.133 on (8, 44), p-value: 0.0525

RBC (optimized model)

	coeff.	s.e.	p-value	
internal x immature	0.00109	0.000725	0.139	
internal x mature	-0.00049	0.00039	0.214	
external x immature	-0.00292	0.00223	0.196	
external x mature	-0.00254	0.00175	0.154	
age	0.00714	0.00362	0.0547	
sex	-	-	-	
season	0.0634	0.0261	0.019	*
altitude	-	-	-	

N=53, Adjusted R^2=0.166, AIC=-100.799
F-statistic: 2.719 on (6, 46), p-value: 0.0241*

"-" indicates unavailable information due to no-use in the model.
WBC: White blood cells, RBC: Red blood cells, coeff.: coefficient, s.e.: standard error.
*: 0.01 ≤ p <0.05, **: 0.001 ≤ p < 0.01, ***: p < 0.001

Supplementary Table S1 (Continued)

Hb (full model)

	coeff.	s.e.	p-value	
internal x immature	0.00053	0.00075	0.488	
internal x mature	-0.00079	0.00040	0.0526	
external x immature	-0.00067	0.00251	0.791	
external x mature	0.00319	0.00317	0.321	
age	0.00050	0.00456	0.914	
sex	0.02080	0.0264	0.436	
season	0.11000	0.0308	0.00098	**
altitude	0.02380	0.0131	0.0768	

N=47, Adjusted R^2=0.259,AIC=-91.263

F-statistic: 3.005 on (8, 38), p-value: 0.0103*

Hb (optimized model)

	coeff.	s.e.	p-value	
internal x immature	0.00065	0.00072	0.371	
internal x mature	-0.00074	0.000381	0.0595	
external x immature	-0.00093	0.00243	0.704	
external x mature	0.00305	0.00254	0.238	
age	-	-	-	
sex	-	-	-	
season	0.117	0.0285	0.0002	***
altitude	.0246	.0103	0.0222	*

N=47, Adjusted R^2=0.284, AIC=-94.480

F-statistic: 4.038 on (6, 40), p-value: 0.00296**

Hct (full model)

	coeff.	s.e.	p-value	
internal x immature	0.00043	0.000716	0.557	
internal x mature	-0.00029	0.000376	0.444	
external x immature	0.00051	0.0024	0.833	
external x mature	0.00184	0.00193	0.344	
age	0.00186	0.000321	0.729	
sex	0.0170	0.0252	0.505	
season	0.00020	0.0294	0.995	
altitude	0.0180	0.0125	0.159	

N=47, Adjusted R^2=-0.0005, AIC=-95.519

F-statistic: 0.997 on (8, 38), p-value: 0.454

Hct (optimized model)

	coeff.	s.e.	p-value	
internal x immature	-0.0005	0.00065	0.452	
internal x mature	-0.00023	0.00035	0.526	
external x immature	0.000322	0.00220	0.884	
external x mature	0.00336	0.00240	0.168	
age	-	-	-	
sex	-	-	-	
season	-	-	-	
altitude	0.021	0.00976	0.0371	*

N=47, Adjusted R^2=0.0559, AIC=-100.677

F-statistic: 1.545 on (5, 41), p-value: 0.197

PLT (full model)

	coeff.	s.e.	p-value	
internal x immature	-0.00288	0.00284	0.317	
internal x mature	-0.0045	0.00152	0.00497	**
external x immature	-0.00197	0.00911	0.83	
external x mature	-0.00388	0.00853	0.652	
age	-0.00089	0.0163	0.957	
sex	-0.0370	0.0972	0.706	
season	0.119	0.103	0.255	
altitude	-0.00474	0.0457	0.918	

N=53, Adjusted R^2=0.135, AIC=43.405

F-statistic: 2.011 on (8, 44), p-value: 0.0673

PLT (optimized model)

	coeff.	s.e.	p-value	
internal x immature	-0.00314	0.00268	0.247	
internal x mature	-0.00452	0.00144	0.00293	**
external x immature	-0.00079	0.00699	0.911	
external x mature	0.000858	0.107	0.994	
age	-	-	-	
sex	-	-	-	
season	0.110	0.097	0.261	
altitude	-	-	-	

N=53, Adjusted R^2=0.187, AIC=37.60

F-statistic: 3.388 on (5, 47), p-value: 0.0107*

"-" indicates unavailable information due to no-use in the model.

Hb: Hemoglobin, Hct: Hematocrit, PLT: Platelets, coeff.: coefficient, s.e.: standard error.

*: 0.01 ≤ p <0.05, **: 0.001 ≤ p < 0.01, ***: p < 0.001

Supplementary Table S2

Supplementary Table S2. Correlation coefficients between haematological values in the bone marrow and dose-rates.

	Internal dose-rate		External dose-rate		Total dose-rate	
	r	p	r	p	r	p
Mature (18)						
Erythroid	-0.246	0.297	-0.191	0.419	-0.245	0.298
Myeloid	-0.428	0.060	-0.238	0.312	-0.401	0.080
Megakaryocyte	-0.033	0.890	0.443	0.051	0.096	0.686
Haematopoietic cells	-0.549	0.012	-0.288	0.219	-0.509	0.022
Adipose tissue	0.585	0.007	0.240	0.307	0.525	0.018
Immature (20)						
Erythroid	0.214	0.366	0.112	0.637	0.207	0.381
Myeloid	-0.036	0.881	-0.472	0.036	-0.133	0.577
Megakaryocyte	0.148	0.533	-0.042	0.861	0.118	0.621
Haematopoietic cells	-0.112	0.639	-0.460	0.041	-0.196	0.409
Adipose tissue	0.149	0.530	0.225	0.341	0.177	0.456

r and p indicate pearson’s correlation coefficient and p value, respectively.
The numbers in parentheses indicate the number of animals examined.

Supplementary Table S3

Supplementary Table S3: Results of multiple regression analyses for the haematopoieitic values in bone marrow of macaques with covariates sex, age, season of capture date and altitude of capture: Estimated regression coefficients and goodness of fit using the full model and the optimized model.

Erythroid cells (full model)

	coeff.	s.e.	p-value	
internal x immature	0.0014	0.00142	0.333	
internal x mature	-0.00323	0.00343	0.353	
external x immature	-0.00058	0.00665	0.931	
external x mature	0.00504	0.0108	0.643	
age	-0.023	0.0219	0.302	
sex	-0.0255	0.0755	0.738	
season	0.0324	0.0771	0.677	
altitude	-0.00131	0.00248	0.601	

N=38, Adjusted R^2=-0.0291, AIC=-8.036
F-statistic: 0.869 on (8, 29), p-value: 0.553

Erythroid cells (optimized model)

	coeff.	s.e.	p-value	
internal x immature	0.00117	0.0013	0.373	
internal x mature	-0.00268	0.00309	0.393	
external x immature	-0.00303	0.00491	0.542	
external x mature	0.0018	0.00858	0.853	
age	-0.0232	0.0205	0.266	
sex	-	-	-	
season	-	-	-	
altitude	-	-	-	

N=18, Adjusted R^2=0.0549, AIC=13.532
F-statistic: 1.43 on (5, 32), p-value: 0.240

Myeloid cells (full model)

	coeff.	s.e.	p-value	
internal x immature	0.00155	0.000892	0.0933	
internal x mature	-0.00528	0.00215	0.0202	*
external x immature	-0.00663	0.00417	0.123	
external x mature	0.00897	0.00675	0.194	
age	-0.0338	0.0137	0.0196	*
sex	0.00847	0.0473	0.859	
season	0.0448	0.0483	0.362	
altitude	-0.169	0.156	0.287	

N=38, Adjusted R^2=0.277, AIC=-43.566
F-statistic: 2.772 on (8, 29), p-value: 0.0208*

Myeloid cells (optimized model)

	coeff.	s.e.	p-value	
internal x immature	0.00142	0.000834	0.0989	
internal x mature	-0.00506	0.00198	0.0157	*
external x immature	-0.00977	0.00315	0.00398	**
external x mature	0.00656	0.0055	0.242	
age	-0.0363	0.0132	0.00958	**
sex	−	−	−	
season	−	−	−	
altitude	−	−	−	

N=38, Adjusted R^2=0.304, AIC=-47.287
F-statistic: 4.237 on (5, 32), p-value: 0.00455

“-” indicates unavailable information due to no-use in the model.
WBC: White blood cells, RBC: Red blood cells, coeff.: coefficient, s.e.: standard error.
*: 0.01 ≤ p <0.05, **: 0.001 ≤ p < 0.01, ***: p < 0.001

Supplementary Table S3 (Continued)

Megakaryocyte (full model)

	coeff.	s.e.	p-value	
internal x immature	0.000283	0.00144	0.846	
internal x mature	-0.00725	0.00347	0.0454	*
external x immature	0.00348	0.00673	0.609	
external x mature	0.0262	0.0109	0.0226	*
age	-0.0445	0.0221	0.0537	
sex	0.175	0.0763	0.029	*
season	-0.0188	0.078	0.811	
altitude	-0.233	0.251	0.361	

N=38, Adjusted R^2=0.260, AIC=-7.173
F-statistic: 2.621 on (8, 29), p-value: 0.0272*

Megakaryocyte (optimized model)

	coeff.	s.e.	p-value	
internal x immature	0.000237	0.00139	0.865	
internal x mature	-0.00717	0.00335	0.0401	*
external x immature	0.000168	0.00512	0.974	
external x mature	0.0212	0.00944	0.032	*
age	-0.0436	0.0217	0.0531	
sex	0.188	0.073	0.0152	*
season	-	-	-	
altitude	-	-	-	

N=38, Adjusted R^2=0.279, AIC=-9.641
F-statistic: 3.385 on (6, 31), p-value: 0.011*

Haematopoietic cells (full model)

	coeff.	s.e.	p-value	
internal x immature	0.000505	0.000609	0.414	
internal x mature	-0.005	0.00147	0.00191	**
external x immature	-0.00401	0.00284	0.17	
external x mature	0.00686	0.00461	0.148	
age	-0.0265	0.00935	0.0082	**
sex	-0.0133	0.0323	0.684	
season	-0.0156	0.033	0.64	
altitude	-0.0251	0.106	0.815	

N=38, Adjusted R^2=0.376, AIC=-72.599
F-statistic: 3.789 on (8, 29), p-value: 0.00375**

Haematopoietic cells (optimized model)

	coeff.	s.e.	p-value	
internal x immature	0.00049	0.000558	0.386	
internal x mature	-0.00493	0.00133	0.000763	***
external x immature	-0.0042	0.00211	0.0547	
external x mature	0.0057	0.0037	0.131	
age	-0.0253	0.00881	0.0071	**
sex	-	-	-	
season	-	-	-	
altitude	-	-	-	

N=38, Adjusted R^2=0.424, AIC=-77.856
F-statistic: 6.436 on (5, 32), p-value: 0.0003***

Adipose tissue (full model)

	coeff.	s.e.	p-value	
internal x immature	-2E-06	0.00084	0.999	
internal x mature	0.00689	0.002	0.00196	**
external x immature	-0.00194	0.00393	0.626	
external x mature	-0.0173	0.00637	0.0109	*
age	0.0229	0.0129	0.0865	
sex	0.0195	0.0446	0.665	
season	0.0114	0.0456	0.8505	
altitude	0.2481	0.1468	0.102	

N=38, Adjusted R^2=0.205, AIC=-47.997
F-statistic: 2.193 (8, 29), p-value: 0.05828*

Adipose tissue (optimized model)

	coeff.	s.e.	p-value	
internal x immature	0.000049	0.00078	0.95	
internal x mature	0.00672	0.00185	0.00102	**
external x immature	-0.00205	0.00357	0.57	
external x mature	-0.0164	0.00571	0.00728	**
age	0.0216	0.0123	0.09	
sex	-	-	-	
season	-	-	-	
altitude	0.247	0.132	0.0714	

N=38, Adjusted R^2=0.249, AIC=51.610
F-statistic: 3.041 (6, 31), p-value: 0.0186*

"-" indicates unavailable information due to no-use in the model.
Hb: Hemoglobin, Hct: Hematocrit, PLT: Platelets, coeff.: coefficient, s.e.: standard error.
*: $0.01 \leq p < 0.05$, **: $0.001 \leq p < 0.01$, ***: $p < 0.001$

Supplementary Table S4

Supplementary Table S4. Dose conversion coefficient of each spheroid

Height cm	Width* cm	Length* cm	Mass* kg	Internal DCC (μGy/day)/(Bq/kg)		External DCC (μGy/day)/(Bq/m^2)	
				^{134}Cs	^{137}Cs	^{134}Cs	^{137}Cs
30	9.3	9.3	1.4	5.41E-3	4.59E-3	1.16E-4	4.25E-5
40	12.7	12.7	3.4	6.46E-3	4.98E-3	1.10E-4	3.98E-5
50	16.1	16.1	6.8	7.47E-3	5.36E-3	1.04E-4	3.79E-5
60	19.5	19.5	12.0	8.46E-3	5.72E-3	9.70E-5	3.53E-5

*Values of Width, Length and Mass were calculated (see the dose-rate estimation part of the Methods section).
